# Reporting of Discrimination by Health Care Consumers Through Online Consumer Reviews

**DOI:** 10.1001/jamanetworkopen.2022.0715

**Published:** 2022-02-28

**Authors:** Jason K. C. Tong, Eda Akpek, Anusha Naik, Medha Sharma, Danielle Boateng, Anietie Andy, Raina M. Merchant, Rachel R. Kelz

**Affiliations:** 1 National Clinician Scholars Program, Michael J. Crescenz Veterans Affairs Medical Center, Philadelphia, Pennsylvania; 2 Center for Surgery and Health Economics, Hospital of the University of Pennsylvania, Philadelphia; 3 Department of Surgery, Perelman School of Medicine, University of Pennsylvania, Philadelphia; 4 Leonard Davis Institute of Health Economics, Philadelphia, Pennsylvania; 5 Department of Surgery, Perelman School of Medicine, University of Pennsylvania, Philadelphia; 6 Penn Mixed Methods Research Laboratory, Perelman School of Medicine, University of Pennsylvania, Philadelphia; 7 Center for Digital Health, Perelman School of Medicine, University of Pennsylvania, Philadelphia; 8 Department for Emergency Medicine, Perelman School of Medicine, University of Pennsylvania, Philadelphia

## Abstract

**Importance:**

Little is known about how discrimination in health care relates to inequities in hospital-based care because of limitations in the ability to measure discrimination. Consumer reviews offer a novel source of data to capture experiences of discrimination in health care settings.

**Objective:**

To examine how health care consumers perceive and report discrimination through public consumer reviews.

**Design, Setting, and Participants:**

This qualitative study assessed Yelp online reviews from January 1, 2011, to December 31, 2020, of 100 randomly selected acute care hospitals in the US. Word filtering was used to identify reviews potentially related to discrimination by using keywords abstracted from the Everyday Discrimination Scale, a commonly used questionnaire to measure discrimination. A codebook was developed through a modified grounded theory and qualitative content analysis approach to categorize recurrent themes of discrimination, which was then applied to the hospital reviews.

**Exposures:**

Reported experiences of discrimination within a health care setting.

**Main Outcomes and Measures:**

Perceptions of how discrimination in health care is experienced and reported by consumers.

**Results:**

A total of 10 535 reviews were collected. Reviews were filtered by words commonly associated with discriminatory experiences, which identified 2986 reviews potentially related to discrimination. Using the codebook, the team manually identified 182 reviews that described at least 1 instance of discrimination. Acts of discrimination were categorized by actors of discrimination (individual vs institution), setting (clinical vs nonclinical), and directionality (whether consumers expressed discriminatory beliefs toward health care staff). A total of 53 reviews (29.1%) were coded as examples of institutional racism; 89 reviews (48.9%) mentioned acts of discrimination that occurred in clinical spaces as consumers were waiting for or actively receiving care; 25 reviews (13.7%) mentioned acts of discrimination that occurred in nonclinical spaces, such as lobbies; and 66 reviews (36.3%) documented discrimination by the consumer directed at the health care workforce. Acts of discrimination are described through 6 recurrent themes, including acts of commission, omission, unprofessionalism, disrespect, stereotyping, and dehumanizing.

**Conclusions and Relevance:**

In this qualitative study, consumer reviews were found to highlight recurrent patterns of discrimination within health care settings. Applying quality improvement tools, such as the Plan-Do-Study-Act cycle, to this source of data and this study’s findings may help inform assessments and initiatives directed at reducing discrimination within the health care setting.

## Key Points

**Question:**

Can publicly available consumer reviews highlight experiences of discrimination in hospital-based health care delivery and offer strategies for improvement?

**Findings:**

In this qualitative study, content analysis of consumer reviews of US acute care hospitals revealed recurrent themes of discrimination directed toward health care consumers not otherwise available in traditional hospital performance metrics. Analysis also highlighted evidence of discrimination directed from consumers toward health care staff.

**Meaning:**

Further work correlating these subjective reports of discrimination with objective measures of inequities in care may help to develop a health care–specific measure of discrimination to aid in its study and eradication.

## Teaser

This qualitative study of health care consumers examines reporting of discrimination in hospital-based health care delivery through public consumer reviews.

## Introduction

Health inequities exist across disciplines and patient characteristics.^1,2^ Although outdated models focused on biological drivers of inequities, newer studies^3,4,5^ have shifted attention toward structural and interpersonal discrimination’s primary role in driving inequities. Many studies^6,7,8,9,10,11^ have demonstrated how discrimination propagates worse health outcomes for minority populations across race, sex, gender, sexual orientation, age, and disability. Consequently, there are increasing efforts in identifying and reducing experiences of discrimination within health care.^12,13^

Attempts at studying discrimination’s role in driving inequities are limited by a lack of available tools to measure discrimination within health care.^14^ Traditional health care performance metrics, such as Hospital Compare, do not report on discrimination or health inequities.^15^ Alternative qualitative tools, such as the Index of Race-Related Stress,^16^ are effective in smaller settings and are validated to capture a variety of discriminatory experiences; however, in their existing format, these tools are difficult and costly to administer on larger scales, refer to lifelong experiences rather than time-limited health care encounters, and are limited by low response rates, potentially because of fear of retaliation.

Analyzing publicly available online consumer reviews offers a potential approach to identifying experiences of discrimination in health care. Consumer reviews have been shown to correlate with traditional performance metrics and highlight novel aspects of the patient experience otherwise not captured through existing measures.^17^ Additional advantages include consumer reviews’ unsolicited and anonymous nature, increasing influence on consumer health care choices, and timely availability, which allows for more prompt analysis of the consumer voice.^18,19^ In addition, studies^20,21^ have found that consumer reviews and social media serve as an attractive source of information on health care and that minority individuals, such as Black health care consumers, rely on digital media to guide their health care decisions.

In response to the need for additional approaches to collecting information on discrimination in health care, we performed a qualitative study of unsolicited online consumer reviews. We aimed to reveal how consumers experience and report on discrimination during hospital-based care through qualitative content analysis. We hypothesized that qualitative content analysis of consumer reviews would yield recurring themes of discrimination to inform future efforts in measuring discrimination in health care.

## Methods

### Settings, Participants, and Study Design

We selected Yelp to perform this quantitative study because it is widely used, is updated frequently, and uses a proprietary algorithm to filter potentially falsified reviews.^17,22^ The demographic characteristics and identities of the consumer review authors were not abstracted for analysis, and no review authors were contacted. This study was deemed exempt from review by the University of Pennsylvania Institutional Review Board. This study followed the Strengthening the Reporting of Observational Studies in Epidemiology (STROBE) and Standards for Reporting Qualitative Research (SRQR) reporting guidelines.^23,24^

Hospitals with review webpages deemed “not recommended” by Yelp’s proprietary algorithm that filters potentially falsified pages and reviews were excluded in accordance with prior work.^22,25^ We then randomly sampled 100 acute care hospitals with at least 10 published reviews from a list of facilities identified as hospitals by Yelp (n = 7885). We then abstracted hospital characteristics for these 100 hospitals by manually reviewing each hospital’s reported website to determine the American Hospital Association survey data set facility name and linked the 2 data sources. If a facility name was not an exact match, the address was then matched by 1 of us (J.K.C.T.). Hospital reviews published between January 1, 2011, and December 31, 2020, were collected for study.

### Review Filtering and Codebook Development

Through prior experience exploring racism in online reviews, we anticipated a large amount of data associated with our 100 randomly selected hospitals, most of which would likely be unrelated to discrimination.^26^ We used the original version of the Everyday Discrimination Scale, a widely used and validated questionnaire for studying discrimination, as a conceptual framework.^27,28^ We abstracted 31 keywords and synonyms from the questionnaire related to how individuals experience discrimination. We applied a word filter to collect reviews that contained any of the 31 keywords to identify reviews most likely related to discrimination. See eTable 1 in the Supplement for a list of keywords.

The coding team consisted of a postgraduate year 5 general surgery resident (J.K.C.T.), medical students trained in qualitative coding (A.N., M.S., and D.B.), and a research coordinator formally trained in qualitative methods (E.A.). The codebook was developed using a separate training set of collected reviews defined by a purposive sample of well-known and highly ranked hospitals according to the *US News and World Report* top-ranking hospital list during a 10-year period.^29^ This sample was defined in this manner with the thought that the most widely publicized hospitals would be associated with the highest frequency of reviews. In total, 32 hospitals were identified, and half were randomly selected for codebook development. Our keyword filter was applied to all reviews associated with those hospitals, yielding a sample of 340 reviews. Following the initial codebook development, these reviews were excluded for subsequent qualitative content analysis to avoid potential bias given the likely familiarity of these institutions by content coders.

The developed codebook assigned acts of discrimination to an individual actor or group of individual actors (institution), categorized reviews by their setting to clinical or nonclinical contexts (such as billing departments), and labeled reviews in which consumers expressed discriminatory views toward health care staff as health care directed (Figure). After coding reviews by their actors, setting, and directionality, the coding team applied a modified grounded approach to the Everyday Discrimination Scale and coded reviews to generate 6 broad, comprehensive categories of how discrimination manifests and is experienced by consumers (Table 1).

**Figure.**
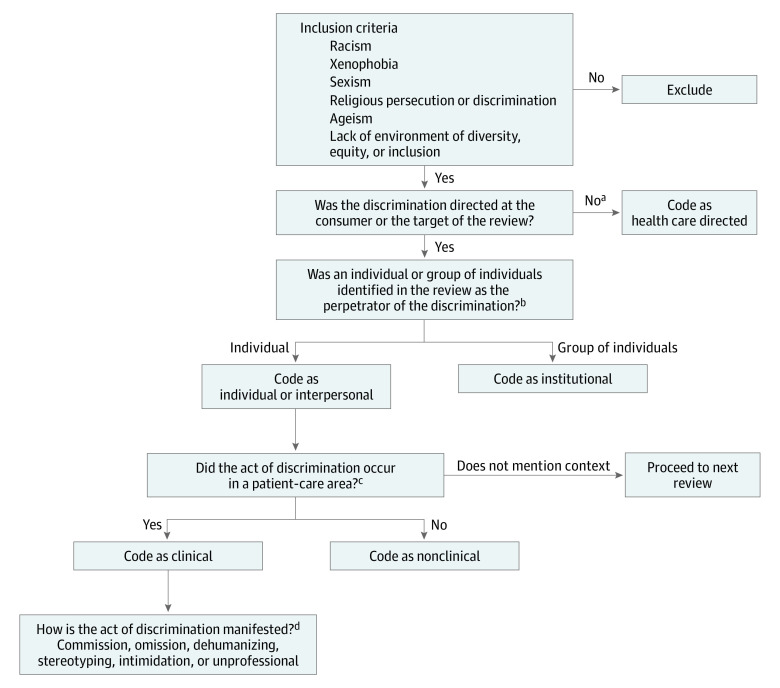
Discrimination Codebook Coding algorithm used by coders to abstract recurrent ideas, including actors, setting, directionality, and pattern of manifestation. ^a^If review includes discrimination by the consumer toward health care, then code as health care directed. ^b^Reviews should only be coded as individual or interpersonal or as institutional rather than double coding (do not double code). If both institutional and individual sources are mentioned, code as institutional. ^c^Reviews may make mention of acts of discrimination occurring in multiple places (clinical and nonclinical). Both should be coded as such (okay to double code). ^d^Act of commission: consumer reports additional measures performed because of discrimination, including verbal and physical abuse. Act of omission: consumer reports withholding of care or resources because of discrimination, including lack of privacy. Dehumanizing: explicitly states individual or institution as devaluing or dehumanizing consumer. Key terms include *humiliate*, *objectify*, *embarrass*, or *devalue*. Stereotyping: states reviewer is stereotyped or scapegoated. Describes the use of stereotypes during encounter. Intimidation: reviewer mentions being scared, afraid, or frightened because of acts of individual’s or institution’s discrimination. Unprofessional: explicitly states individuals or institutions as disrespectful, unprofessional, or rude. May include mention of hospital environment lacking in diversity or professionalism.

**Table 1.** Manifestations of Discrimination and Coding Definitions

Manifestation of discrimination^a^	Definition
Act of omission	Consumer reports withholding of care or resources because of discrimination, including lack of privacy
Act of commission	Consumer reports additional measures performed because of discrimination, including instances of verbal or physical abuse
Disrespectful or unprofessional	Explicit mention of individuals or institution as disrespectful, unprofessional, or rude; may also include mention of a hospital environment lacking in diversity or professionalism
Devaluation or dehumanizing	Explicit mention of individuals or institutions as devaluing or dehumanizing; key terms such as *humiliate*, *objectify*, *embarrass*, or *devalue* may be used
Scapegoating or stereotyping	Consumer reports an individual is being stereotyped or scapegoated; must make mention of specific stereotype (“name-calling” or “slurs” is not sufficient)
Intimidation	Consumer mentions being scared, afraid, or frightened because of the acts of an institution’s or individual’s discrimination

^a^ These 6 manifestations of discrimination were generated through group discussion and application of modified grounded theory to the original version of the Everyday Discrimination Scale.

After manual iterative review and content coding, the coding team reconciled coding discrepancies through group discussion and consensus. All 5 coders (J.K.C.T., E.A., A.N., M.S., and D.B.) were involved in the codebook development and coding reconciliation process. After achieving an adequate group interrater reliability κ score of 0.70 to ensure consistent content coding across coders, the remaining reviews were divided and coded in preset coder dyads. A final group interrater reliability κ score of 0.78 was obtained. See eTable 2 in the Supplement for assigned data sets by coding dyads. Yelp data were first collected and managed in Microsoft Excel, version 16.5 (Microsoft Corp) and subsequently transferred to NVIVO, version 1.4 (QSR International), a qualitative analysis software, for content coding and κ score calculation.

## Results

A total of 10 535 reviews were identified. Table 2 lists the hospital characteristics. Keyword filtering identified 2986 reviews potentially related to discrimination. Numbers of filtered reviews per hospital ranged from 0 to 124. There were 182 reviews that signaled discrimination, with 72 (39.6%) citing individual actors. These actors varied from volunteer greeters to security officers, nurses, and physicians. Reviews varied in the details offered regarding acts of discrimination. Some consumers identified actors’ actions as discriminatory, whereas others offered more explicit detail on how specific hospital employees acted in a biased manner based on consumer attributes, such as race or gender. A total of 53 of 182 reviews (29.1%) were coded as examples of institutional racism; 89 reviews (48.9%) mentioned acts of discrimination that occurred in clinical spaces as consumers were waiting for or actively receiving care; 25 reviews (13.7%) mentioned acts of discrimination that occurred in nonclinical spaces, such as lobbies; and 66 reviews (36.3%) documented discrimination by the consumer directed at the health care workforce. After coding reviews by their actors, setting, and directionality, we identified that acts of discrimination are described and manifested in 6 recurring patterns: acts of commission, omission, dehumanizing, stereotyping, intimidation, and unprofessionalism.

**Table 2.** Characteristics of Study Hospitals With and Without Reviews Reporting Discrimination^a^

Hospital characteristic	No. (%) of hospitals		
	Total (N = 89)	Hospitals with reviews reporting discrimination (n = 58)	Hospitals without reviews reporting discrimination (n = 31)
Hospital size, No. of beds			
6-49	4 (4.5)	3 (5.2)	1(3.2)
50-199	35 (39.3)	22 (37.9)	12 (38.7)
200-399	33 (37.1)	23 (39.7)	10 (32.3)
≥400	17 (19.1)	10 (17.2)	8 (25.8)
Region			
Northeast	14 (15.7)	7 (12.1)	6 (19.4)
South	32 (36.0)	19 (32.8)	14 (45.2)
Midwest	3 (3.4)	2 (3.4)	1 (3.2)
West	40 (44.9)	30 (51.7)	10 (32.3)
Teaching hospital	79 (88.8)	54 (93.1)	25 (80.6)
Ownership			
Government			
Nonfederal	8 (9.4)	5 (9.1)	3 (10.0)
Federal	3 (3.5)	3 (5.5)	0
Nongovernment, nonprofit	62 (72.9)	38 (69.1)	24 (80.0)
Investor owned, for profit	12 (14.1)	9 (16.4)	3 (10.0)

^a^ Validated Centers for Medicare & Medicaid Services identification was available for 11 hospitals but was not present within the American Hospital Association data set. Coded hospitals include those with at least 1 review coded to a theme of discrimination.

### Manifestations of Discrimination

#### Act of Commission

Acts of commission involved instances in which actors showed their biases through purposeful acts of physical or verbal harassment. In extreme examples, a few reviews mentioned instances when actors violated patients’ consent in carrying out abuses: “When will we women stop being sexually harassed. You’d think you could see a medical professional without that happening.” As a result, consumers believed that they were receiving differential treatment, particularly when actors prevented or made it difficult to engage with the health care system, such as when seeking care or visiting family members: “The nurses on [unit] are extremely racist. One of the nurses literally said, ‘You people,’ and said that we frightened her so much that she was afraid to leave her job…. They had security escort me off the hospital floor and said I could no longer visit my mom. EVER.” Reviews noted some actors justified their discriminatory behavior using hospital policies. Many consumer reviewers feared that filing a grievance would be met with retribution, such as by making notes in the patient’s medical record to affect care: “Most importantly do not make a…nurse an enemy by filing a grievance with member services because they will go through your chart and tell the doctors how to treat you for retribution.”

#### Act of Omission

Acts of omission described instances in which medical care or basic needs, such as food or assistance with activities of daily living, were neglected or delayed by hospital staff. These reviews highlighted the unequal balance of power perceived by consumers. Many reviews revealed that these behaviors continued despite engagement by patients and their advocates: “If you’re thinking about having a baby here, don’t. Especially if you’re a black woman. The racism I witnessed…. They made my cousin wait 2 hours…. And the nurse who was rude to her throughout her whole entire labor told her ‘there are more important people to take care of’.” Acts of omission frequently manifested around discussions of pain. Consumers described how a lack of attention to pain ultimately led to a missed or delayed diagnosis of an acute medical issue that was only discovered after seeking second or third opinions. At times, unequal power dynamics seemed further amplified when several forms of discrimination were present simultaneously. Some consumers believed that these acts of omission and delays in care were deliberate on the part of health care organizations to prolong treatment and increase financial gain, further demonstrating a sense of mistrust of health care institutions by certain patient populations: “My pain level, symptoms and medication history were mis-recorded resulting in poor pain management, and a misdiagnosis that would have resulted in a complicated, unnecessary surgery had I not signed myself out for a second opinion…the nurses and doctors were sexist and homophobic, hostile to my partner and family.”

#### Dehumanizing

Dehumanizing manifestations portrayed the consumer feeling dehumanized or devalued compared with others because of a particular personal attribute. For example, one consumer wrote, “Why wasn't I greeted with enthusiasm, let alone greeted at all? Was it because of the color of my skin? Am I less of a person? Or was it because of age discrimination? In all my time here in the healthcare systems in [city], I’ve never once felt this invalidated.” Most frequently, consumers reported feeling dehumanized because of being ignored in a variety of settings. In some instances, consumers felt ignored because of a lack of communication attributed to poor staff training on same-sex couples, whereas in others, consumers cited instances in which their older relatives were dehumanized by a lack of attention to basic human rights or needs: “A staff member completely opened my [grandmother’s] gown in front of a male transport that didn’t even work for the hospital. Apparently since she is too old to know better it’s okay to show her body to anyone and show her no respect. If you have a choice go somewhere else.” The consumer frequently went on to describe how these acts were so degrading and traumatic that they would never return to that institution.

#### Stereotyping

Consumers often reported on racial and gender stereotypes that perpetuated poor health care treatment, including dismissal of symptoms and pain severity. In these scenarios, the patient came to the practitioner seeking treatment, only for their symptoms to be overlooked because of the practitioners’ prejudices and biases. These experiences occurred often among self-identified Black people and women. For example, self-reported female African American consumers highlighted how some physicians ignored their pain, potentially because of stereotypes of drug-seeking behaviors by people of certain ethnicities, but were also described as anxious or hysterical because of their female gender: “After hearing me complain to my husband that something was not right and that I was still in pain, the nurse came in and asked, ‘What do you want, pain medication?’ As if I was a drug addict only there to get my fix. At this moment I realized I was being judged, singled out, possibly because of my ethnicity, it being late at night and in my pajamas.” Other instances of stereotyping occurred as microaggressions, such as expressing biased opinions, making assumptions about patients, or thoughtlessly saying ignorant statements: “When one of the nurses put on a face mask, a doctor asked her, ‘Are you [going] Islam on me?’ He thought it was funny and laughed about it. I thought this was very unprofessional.”

#### Intimidation

Intimidation manifested as verbal and physical tactics used by health care workers, such as threats of using specific medical protocols as punishments or intrusions into consumers’ personal space, to bully and harass consumers during health care visits. Consumers reported being frightened by individual or institutional discrimination. Frequently, acts of intimidation occurred during psychiatric visits and toward self-identified women or older adults: “He was insisting that I undress in front of him…. He said to me it’s ‘what he has to do[,] all patients have to put on a hospital gown.’” Consumers also described how that intimidation compounded the stress and anxiety of being in a health care setting, especially during an emergency.

#### Unprofessional

Discrimination described as unprofessional manifested as disrespectful or unprofessional behaviors, often including terms such as *mean*, *rude*, and *condescending*. In addition, several consumers noted that unprofessional individuals shared personal thoughts and opinions that expressed bias, judgment, microaggression, and macroaggression. In such instances, respondents believed that perpetrators’ negative attitude and treatment was caused by bias and in violation of the standard of care. Several offered that such staff should undergo cultural competency training and that being courteous, compassionate, and affirming patients’ conditions and needs are important aspects of professionalism: “We are a married, same-sex couple who were overjoyed to welcome our first son, but that joy was quickly overshadowed by the behavior of some, if not most of the staff. Most of the nurses and any doctor we saw basically completely refused to acknowledge my presence…. I sincerely hope that other couples have a more positive experience and that some steps will be taken to educate staff on how to compassionately handle situations of this nature.”

## Discussion

This exploratory qualitative content analysis of consumer reviews highlights the pervasive nature of discrimination in medicine through its (1) presence in clinical and nonclinical spaces, (2) display by individual and institutional actors, and (3) multidirectional flow between health care consumers and practitioners. This work builds on a previous study^30^ of reviews mentioning racism by examining consumer reviews for the broader categories of discrimination most described in the literature. Similar to prior work,^26,31,32,33^ we found that discrimination is perpetrated by not only individual health care practitioners but also entire institutions as mediated by specific policies or groups of people. In addition, we identified 6 recurrent themes that reflect the manifestations of discrimination.

For the first time, to our knowledge, we report these 6 manifestations to serve as a conceptual framework for subsequent programs designed to reduce discrimination in health care. Notably, each of the 6 manifestations of discrimination identified within this study have been previously (and independently) described as separate individual drivers of medical errors and lapses in patient safety.^34,35,36,37,38^ Consequently, it stands to reason that these manifestations of discrimination might result in harm to individuals and lead to inequities in care. The ability to identify behaviors that underlie acts of discrimination provides opportunity to develop tools to screen and measure the prevalence of these manifestations,^36,39,40^ allowing for the development of targeted interventions. For example, acts of commission and omission, often described as processes that lead to medical errors,^41,42^ have been targeted in efforts to reduce medication errors with great success.^43,44^

By approaching discrimination within health care as a form of patient harm, numerous effective quality improvement tools, such as the Plan-Do-Study-Act cycle,^45,46^ become available. By using available data, such as consumer reviews, institutions may perform focused assessments to understand which of the 6 manifestations of discrimination are present within individual institutions. The “plan” phase allows hospitals to better understand what issues of discrimination are present and plan for directed educational efforts to individual and institutional perpetrators on these shortcomings. As some studies highlight,^36,39,40,47,48,49^ most perpetrators of medical error are unaware of these gaps in care and thus require nuanced educational efforts to reorient them on how and why episodes of patient harm occur. Following the implementation of these guided interventions in a “do” phase, hospitals can then measure any potential improvement or exacerbation of discriminatory practices in the “study” phase. Finally, with that data, hospitals can devise plans for the “act” phase, including surveillance and ongoing improvement on any manifestations of discrimination present within the institution.

### Limitations

This study has several limitations. First, although 10 535 reviews were identified for study, only a small percentage explicitly mentioned discrimination. Second, although our word filter refined the data to a more manageable size and successfully identified reviews that pertained to discrimination, it is unclear how many excluded reviews may have potentially reported on discrimination. Third, future work to correlate the prevalence of these subjective findings of discrimination to objective measures of inequities in care are needed. Fourth, the team was unable to reliably report on physical or mental disability. Further efforts are needed to better understand the needs of this population. Fifth, the research team is limited by the inability to contact review authors to better understand the association between author demographic characteristics and reported discrimination or to clarify unsubstantiated claims of discrimination or vague reviews.

## Conclusions

This qualitative study revealed consumer reviews’ potential to highlight experiences of discrimination during hospital encounters. Throughout reviews across a random sample of US acute care hospitals, 6 recurring manifestations of discrimination were identified, allowing for a framework for addressing discrimination through the lenses of patient safety and quality improvement tools.

## Notes

Supplement. **eTable 1.** List of Keywords Abstracted from Everyday Discrimination Scale **eTable 2.** Coder Dyads Dataset Assignments and the Associated Numbers of Institutions and Reviews
