# Supplementary material for: Reporting of Discrimination by Health Care Consumers Through Online Consumer Reviews
Source: JAMA Netw Open. 2022 Feb 28;5(2):e220715. doi: 10.1001/jamanetworkopen.2022.0715 (PMC8886543; doi:10.1001/jamanetworkopen.2022.0715)
Supplement: Supplement. — eTable 1. List of Keywords Abstracted from Everyday Discrimination Scale eTable 2. Coder Dyads Dataset Assignments and the Associated Numbers of Institutions and Reviews [file jamanetwopen-e220715-s001.pdf]

Supplementary Online Content

Tong JKC, Akpek E, Naik A, et al. Reporting of discrimination by health care consumers through online consumer reviews. *JAMA Netw Open*. 2022;5(2):e220715. doi:10.1001/jamanetworkopen.2022.0715

eTable 1. List of Keywords Abstracted from Everyday Discrimination Scale

eTable 2. Coder Dyads Dataset Assignments and the Associated Numbers of Institutions and Reviews

This supplementary material has been provided by the authors to give readers additional information about their work.

© 2022 Tong JKC et al. *JAMA Network Open.*

**eTable 1.** List of Keywords Abstracted from Everyday Discrimination Scale
Racist, racism, discrim-*, race, black, African-American, nig***, courteous, rude, scared, dishonest, inferior, superior, insulted, condescending, threat, hate, harass, crack**, chin*, white, unfair, discouraged, slur, unprofessional, bias, fear, disrespect, delay, slow, ignored

© 2022 Tong JKC et al. *JAMA Network Open.*

eTable 2. Coder Dyads Dataset Assignments and the Associated Numbers of Institutions and Reviews

Coders Assigned	Sample Number	Number of Hospitals	Number of Reviews
E.A., J.T.	1	20	1,133
E.A., J.T.	2	20	644
A.N., M.S.	3	20	842
D.B., J.T.	4	20	278
D.B., J.T.	5	20	89

J.T. is a general surgery resident trained in qualitative research, A.N., D.B., and M.S. were research assistants trained in qualitative analytic techniques, and E.A. is a qualitative research expert.

© 2022 Tong JKC et al. *JAMA Network Open.*
